# Difference in Susceptibility Between Carbapenemase- and Non–Carbapenemase-Producing Carbapenem-Resistant Enterobacteriaceae

**DOI:** 10.1017/ash.2021.136

**Published:** 2021-07-29

**Authors:** Justin Clark, David Burgess

## Abstract

**Background:** Carbapenem-resistant Enterobacteriaceae (CRE) remain among the most urgent infectious threats according to the CDC Threats Reports. Although focus has often been placed on carbapenemase-producing phenotypes, there is increasing interest in distinguishing the optimal treatment and outcomes of carbapenemase-producing (CP) and non–carbapenemase-producing (NCP) CRE. We compare antimicrobial susceptibility patterns between CP-CRE and NCP-CRE isolated from patients at our academic medical center. **Methods:** All CRE isolates of *Enterobacter cloacae*, *Escherichia coli*, *Klebsiella aerogenes*, *K. oxytoca*, and *K. pneumoniae* in adult inpatients from 2010 to 2019 were included in this study. Susceptibility testing was performed using the BD Phoenix Automated System (BD Diagnostics, Sparks, MD). CLSI susceptibility break points were utilized in the susceptibility analyses of all antimicrobials tested. To determine carbapenemase production, isolates resistant only to ertapenem were considered NCP-CRE, and those resistant to both ertapenem and meropenem were considered CP-CRE. Statistical comparisons of susceptibility profiles were performed using either the χ^2^ test or the Fisher exact test. All data preprocessing and statistical analyses were performed using Python software. **Results:** Over the decade, we identified 291 CRE isolates (216 isolates resistant only to ertapenem and 75 resistant to ertapenem and meropenem). The ertapenem-resistant–only phenotype comprises ~66% of the total CRE population and is largely composed of *E. cloacae* (67%). As expected, most β-lactam susceptibilities were negligibly low between the 2 groups; however, other clinically relevant antimicrobials (aminoglycosides, fluoroquinolones, and sulfamethoxazole/trimethoprim) exhibited starkly different susceptibility profiles (*P* value **Conclusions:** These findings suggest that the most predominant CRE phenotype at our institution is not carbapenemase production. Evaluation of outcomes between CP- and NCP-CRE should be pursued further. The large differences in the MIC distributions may lead to differing outcomes for the affected patients.

**Funding:** No

**Disclosures:** None

